# Radial Corrugations of Multi-Walled Carbon Nanotubes Driven by Inter-Wall Nonbonding Interactions

**DOI:** 10.1007/s11671-010-9801-0

**Published:** 2010-09-30

**Authors:** Xu Huang, Wentao Liang, Sulin Zhang

**Affiliations:** 1 Department of Engineering Science and Mechanics, The Pennsylvania State University, University Park, PA 16802, USA

**Keywords:** Carbon nanotubes, Radial corrugation, Quasi-continuum, vdW interactions

## Abstract

We perform large-scale quasi-continuum simulations to determine the stable cross-sectional configurations of free-standing multi-walled carbon nanotubes (MWCNTs). We show that at an inter-wall spacing larger than the equilibrium distance set by the inter-wall van der Waals (vdW) interactions, the initial circular cross-sections of the MWCNTs are transformed into symmetric polygonal shapes or asymmetric water-drop-like shapes. Our simulations also show that removing several innermost walls causes even more drastic cross-sectional polygonization of the MWCNTs. The predicted cross-sectional configurations agree with prior experimental observations. We attribute the radial corrugations to the compressive stresses induced by the excessive inter-wall vdW energy release of the MWCNTs. The stable cross-sectional configurations provide fundamental guidance to the design of single MWCNT-based devices and shed lights on the mechanical control of electrical properties.

## Introduction

The unique combination of mechanical, electronic, and biochemical properties of carbon nanotubes (CNTs) has found a wide range of applications as building blocks in micro(nano)-electro-mechanical systems (MEMS/NEMS) [1-6]. It has been experimentally demonstrated that mechanical deformation of CNTs is generally coupled with significant changes in the electronic and magnetic properties [4,7,8]. In addition, the structural stability of CNT-based devices has become a major concern in their applications. These have motivated continuing experimental [9-11] and numerical studies [12-20] on the stable morphologies of CNTs.

Because of their extremely large in-plane rigidity compared to their out-of-plane bending rigidity [21], graphene shells were frequently observed to undergo isometric deformation, featuring local folds [12,15-18,22-26]. Previous analyses showed that single-walled carbon nanotubes (SWCNTs) may undergo beam or shell buckling under bending [23,25], compression [23], or twisting [23,24], depending on the aspect ratio of the tube. However, due to the presence of inter-wall van der Waals (vdW) interactions, the physical mechanisms governing the instability of multi-walled carbon nanotubes (MWCNTs), composed of concentric graphene shells, appear to be more complex [12,15-18,27]. For example, wave-like periodic ripplings appear in twisted MWCNT [18], and Yoshimura patterns are present in a bent MWCNT [15,17]. It has been elucidated that these unique deformation patterns are driven by the in-plane strain energy release, penalized by the inter-wall vdW energies [16,18,27,28]. Under pure bending or compression, MWCNTs with inter-wall covalent bridges exhibit evolving morphologies [17]. More recently, we have showed that helically arranged diamond pattern appears in thick, uniaxially compressed MWCNTs [16]. The helically arranged diamond pattern appears to be a coordinated deformation morphology of the rigid inner walls and compliant outer walls in the MWCNTs.

To date, theoretical analyses of the single MWCNTs have been largely based upon the assumption of perfect circular cross-sections [29,30]. Whereas numerical studies [18,28,31,32] predicted that noncircular cross-sectional shapes may be energetically favorable in certain conditions. For example, when bringing two CNTs into close contact, the contact region of the CNTs is fattened [19,31,33,34] to favor inter-tube adhesion energy. Under hydrostatic pressure, cross-sectional shape transformation of MWCNTs from circular to polygonal configurations was observed [28]. Recent experiments reported that MWCNTs synthesized in the presence of nitrogen are constituted of walls with uniform chirality and their cross-sections are of polygonal shapes rather than circular shapes [35,36]. It has been also observed in the experiments that a narrow inner core remains circular while a wide inner core is elongated with facets. It was argued that the polygonal shapes were stabilized in favor of the inter-wall adhesion energy due to the increased inter-wall commensuration. It was also suspected that polygonization may result from the interlayer thermal contraction upon cooling from the synthesis temperature [35].

Motivated by the experiments, in this article, we employ the quasi-continuum method [14,37] to determine the stable cross-sectional configurations of free-standing MWCNTs with different inter-wall spacings and different radii of innermost walls. We show that both factors play significant roles in regulating the cross-sectional configurations of the MWCNTs. Our simulations show that the cross-sections of the free-standing MWCNTs deviate from the normal circular shape and may be stabilized at polygonal or water-drop-like shapes, depending on the inter-wall spacings. In addition, when the radius of the innermost wall in an MWCNT is beyond a critical value, the cross-sections of the MWCNTs may also be stabilized at polygonal shapes and the extent of cross-section polygonization is even greater. These modeling results agree with prior experimental observations. We attribute the stable corrugated cross-sections to the compressive stress induced by the release of the excessive inter-wall vdW energies in the MWCNTs.

The rest of the paper is organized as follows. "Methodology" section briefly introduces the quasi-continuum method. In "Simulation Results" section, we present our simulation results and elucidate the deformation mechanisms. Conclusion remarks are presented in the last section.

## Methodology

We adopt the modified second-generation Brenner potential [38-40], denoted by MTB-G2, to describe the short-range covalent interactions in MWCNTs, which takes the following form:

(1) $$V_{\text{TB}}=\sum_{i}\sum_{j>i}[V^{R}(r_{ij})-B_{ij}(r)V^{A}(r_{ij})],$$

where *r*_*ij*_ is the distance between atoms *i* and *j*, *V*^*R*^ and *V*^*A*^ are the pairwise repulsive and attractive interactions, respectively, *B*_*ij*_ is the bond-order function that has a complicated dependence on the bond angles and bond lengths involving atoms *i* and *j*. The inter-wall vdW interaction is described by a Lennard–Jones (LJ) potential with the parameters given by Girifalco et al. [41], as

(2) $$V_{\text{LJ}}(r)=\frac{\in }{r_{0}^{6}}\left[\frac{1}{2}\kappa^{6}{\left(\frac{r_{0}}{r}\right)}^{12}-{\left(\frac{r_{0}}{r}\right)}^{6}\right],$$

where *r* is the interatomic distance, κ = 2.7 is a dimensionless constant, *r*_0_ = 1.42 Å is the equilibrium bond length and ∈ = 15.2 eV Å^6^.

All-atom simulations with empirical interatomic potentials have been widely used to study the deformation of CNTs [42]. However, for the study of thick MWCNTs, fully atomistic simulations are computationally very expensive because of the large number of degrees of freedom involved. To improve the computational affordability, fully atomistic models are here coarse-grained via a quasi-continuum method based on the finite crystal elasticity theory for curved crystalline monolayers [14,15]. Within the theoretical framework, the exponential Cauchy-Born rule was proposed to link the kinematics at the atomic and continuum scales:

(3) a=ζ(A),

where ζ is an exponential map [14,43] that transforms the undeformed lattice vector **A** into a deformed one **a**. Through a local approximation of the exponential map [14], the deformed lattice vectors and the angles between two lattice vectors can be analytically represented in terms of the continuum deformation measures of the surface, i.e., **C** and **K**, the stretch and curvature tensors, respectively. Considering a representative unit cell of area $S_{0}=(3\sqrt{3}/2)||A|{|}^{2}$ in the graphene lattice, the kinematic link allows analytically determining the hyperelastic strain energy from the underlying interatomic potentials, as

(4) W=W(C;K)=W[a(C;K); α(C;K)],

where **a** and α represent generic lattice vectors and angles between lattice vectors, respectively. The continuum representation of the covalent binding energy for the walls in an MWCNT subject to the deformation map φ that maps from the undeformed to deformed configurations is

(5) $$E=\sum_{i=1}^{n}\int_{\Omega^{i}}W[C[\phi (X)];\;K[\phi (X)]]\text{d}\Omega^{i},$$

where **X** is a material point in the undeformed configuration, ω^*i*^ is the surface area of *i*th wall in an *n*-walled MWCNT.

Homogenization of the discrete inter-wall vdW energy density between two unit cells gives rise to the vdW energy density, as

(6) $$V_{\text{vdW}}(r)={\left(\frac{2}{S_{0}}\right)}^{2}V_{\text{LJ}}(r).$$

The factor of two on the right-hand side of Eq. 6 comes from the fact that each unit cell contains two nuclei. The nonbonded energy between two neighboring shells is then

(7) $$E_{\text{vdw}}=\frac{1}{2}\sum_{i=1}^{n-1}\int_{\Omega_{0}^{i}}\int_{\Omega_{0}^{i+1}}V_{\text{vdW}}[||\phi (X_{i})-\phi (X_{i+1})||]\;\text{d}\Omega_{0}^{i}\;\text{d}\Omega_{0}^{i+1},$$

where **X**_*i*_ and **X**_*i*+1_ are the two material points that are on the *i*th and (*i* + 1)th shells, respectively, in the MWCNT; $\Omega_{0}^{i}$ and $\Omega_{0}^{i+1}$ are the surfaces of the *i*th and (*i* + 1)th shells, respectively.

Based on the coarse-grained constitutive relations for both the bonding and vdW interactions, the constituent shells of the MWCNTs are discretized by finite elements. As extensively tested [12,14-18,43], the coarse-grained model accurately reproduces atomistic simulations; the computational efficiency is improved by about two orders of magnitude when compared to its atomistic counterpart. It should be pointed out that the quasi-continuum method described here is incapable of studying the deformation of defected CNTs, which has been a topic of active research [38,44-50] for the last decade.

## Simulation Results

In the experiments of Ducati et al. [35], the constituent graphene shells of the synthesized MWCNTs are isochiral and likely either zigzag or armchair walls. The measured inter-wall spacings are 0.355 ± 0.009 nm. To determine the effect of inter-wall spacings on the stable cross-sectional configurations of free-standing MWCNTs, the choice of the MWCNTs to be studied is guided by the experimental settings. Three sets of MWCNTs with isochiral walls, indexed by (5,5)/(10,10)/.../(5*n*,5*n*), (9,0)/(18,0)/.../(9*n*,0), and (2,8)/(4,16)/.../(2*n*,8*n*), are chosen for our studies, where *n* is the number of walls in the MWCNTs. All the MWCNTs are 20 nm long. Based on the tube chirality, the three sets of MWCNTs are characterized as armchair (AC), zigzag (ZG), and chiral (CH) MWCNTs, respectively. For each set, five MWCNTs are chosen for our studies with the numbers of walls being 5, 10, 15, 20, and 25. It is useful to remember that the diameter of an (*a*, *b*) SWCNT is given approximately by $0.0783\sqrt{a^{2}+ab+b^{2}}$ nm. Prior to structural optimization, the initial cross-sections of all the MWCNTs are of circular shape and the inter-wall spacings are 0.339, 0.352, and 0.359 nm for the AC, ZG, and CH MWCNTs, respectively. One notes that the initial inter-wall spacings of ZG and CH MWCNTs are 0.12 and 0.19 nm, respectively, larger than the equilibrium spacing of two graphene sheets (0.34 nm) set by the inter-wall vdW interaction potential described by Eq. 2. To obtain the equilibrium configurations, the MWCNTs are fully relaxed free of any constraints using a limited-memory Broyden-Fletcher-Goldfarb-Shanno (BFGS) algorithm [51].

**Figure 1:**
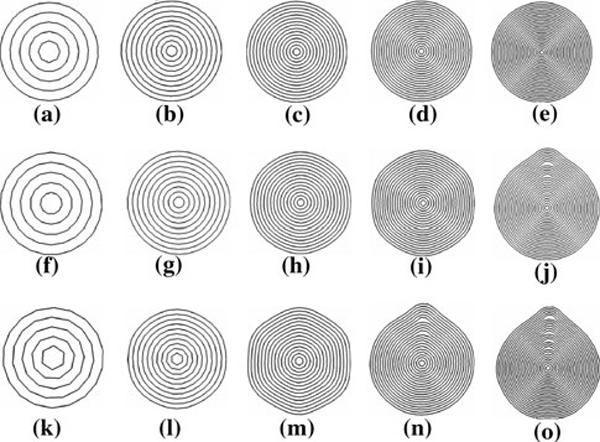
**Cross-sectional views of relaxed MWCNTs**. From *left* to *right* on each row, the wall numbers are 5, 10, 15, 20, and 25. *Top* AC MWCNTs; *middle* ZG MWCNTs; *bottom* CH MWCNTs.

Figure 1 depicts the cross-sectional configurations of relaxed MWCNTs. From top to bottom, the three rows correspond to the AC, ZG, and CH MWCNTs, respectively. Figures on each row from left to right represent the relaxed configurations of 5-, 10-, 15-, 20-, and 25-walled MWCNTs, respectively. It is observed that in the relaxed state, the cross-sections of the AC MWCNTs remain circular shape with graphically invisible morphological change. The cross-sections of the relaxed 5- and 10-walled ZG MWCNT (Figure 1f, g) also remain circular shapes, whereas the 15-walled ZG MWCNT (Figure 1h) is stabilized at a polygonal cross-sectional configuration with 6 rounded corners, which agrees with the experimental observations [35]. Compared to the 15-walled ZG MWCNT, the extent of polygonization of the cross-sections is greater for the 20-walled ZG MWCNT (Figure 1i). For the 25-walled ZG MWCNT, the stabilized cross-section becomes asymmetric, featuring a water-drop-like morphology. For the CH MWCNTs, the cross-sectional shape transformation is even more drastic. Similar trends seen in ZG MWCNTs are observed in CH MWCNTs, except that the polygonized and water-drop-like morphologies occur earlier. From these observations, a general trend of the relaxed cross-section morphologies can be obtained: increasing inter-wall spacings or the number of walls in the MWCNTs drives circular-polygonal-water-drop-like shape transition.

We next elucidate the deformation energetics of the MWCNTs. Regarding the walls as thin shells, the total energy of an MWCNT is the sum of the inter-wall vdW interaction energy, the out-of-plane bending energy, and the in-plane stretching energy. Because of the much higher in-plane stiffness of the graphene walls, in-plane stretching is not an energetically favorable mode. The deformation morphology of an MWCNT free of external loads is a result of the competition between the inter-wall vdW interaction energy and the out-of-plane bending energy. Since the unrelaxed inter-wall spacings for both ZG and CH MWCNTs are higher than the equilibrium inter-wall spacing (0.34 nm) set by the vdW interactions, the excessive inter-wall vdW interaction imposes a compressive stress on the walls. As a result, the MWCNTs are bent to release the inter-wall vdW interaction energy, which drives the polygonization of the cross-sections of the MWCNTs. For the CH MWCNTs, the inter-wall spacing is the largest and so is the driving force for the shape transformation. As a result, the large driving force leads to shape symmetry breaking, and the cross-sections are stabilized at a water-drop-like shape. It should be noted that for the simulations in Figure 1, because of the inner walls are very rigid, it is energetically very costly even to bend these walls. Thus, these inner walls remain circular shapes.

**Figure 2:**
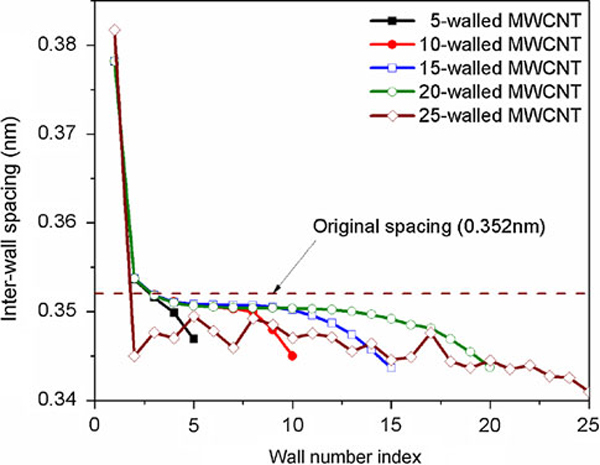
The inter-wall spacing of the relaxed ZG MWCNTs shown in Figure 1 (from (f) to (j)).

Figure 2 shows the average inter-wall spacings of the relaxed configuration of the ZG MWCNTs shown in Figure 1 (from f to j). It should be noted that the spacing of the innermost wall is taken as its radius. Except for the 25-walled MWCNT, the inter-wall spacings of all the other MWCNTs monotonically decrease from inner to outer walls. This is because the outer walls are more compliant, easier to bend, and thus more effective in releasing the inter-wall vdW interaction energy. In contrast, the inter-wall spacings of the 25-walled MWCNT undulate, which may be attributed to the asymmetric cross-sectional corrugation. We also note that for the innermost two walls (except for the 25-walls MWCNT), the relaxed spacings are larger than the original spacing (0.352 nm). This is because of the curvature effect in rolling a graphene layer into the cylindrical shape. Due to the rolling, the bonds become the chords on the curved surface and are shortened. In the relaxed configuration, these bonds are stretched to their equilibrium length in order to minimize the in-plane stretching energy. Thus, the radii of the innermost walls increase. For the rest of the walls, the curvature effects are negligible. Due to the compressive stress generated by the excessive vdW interaction energy release, the inter-wall spacings are reduced to the values lower than the original spacing. One notices from Figure 1f to j that the rounded corners in the relaxed configurations are increasingly more appreciable graphically when the number of walls increases, indicating increasingly higher bending energy. Our analysis thus concludes that the relaxed configuration is a result of inter-wall vdW interaction energy release, penalized by the out-of-plane bending energy.

**Figure 3:**
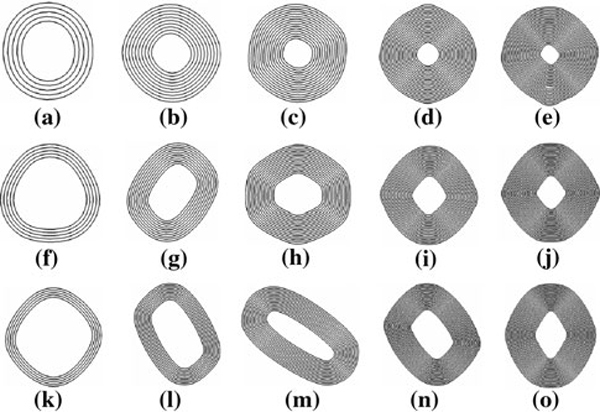
**Cross-sectional views of ZG MWCNTs with different radii of the innermost walls**. From *left* to *right* on each row, the wall numbers are 5, 10, 15, 20, and 25. *Top* ZG MWCNTs with initial, unrelaxed innermost wall radius of 2.1 nm; *middle* ZG MWCNTs with initial, unrelaxed innermost wall radius of 3.8 nm; *bottom* ZG MWCNTs with initial, unrelaxed innermost wall radius of 5.6 nm.

Previous simulations have demonstrated that the innermost walls in thick MWCNTs act as a hard core that plays an important role in the deformation morphologies of MWCNTs [16-18,28]. This explains well the experimental observations that an inner core of small radius remains circular. In order to study the effect of the innermost walls on the stable cross-sectional configurations of free-standing MWCNTs, we considered three sets of ZG MWCNTs with removed innermost walls, thereby varying the radius of the innermost wall of the remaining MWCNTs, as shown in Figure 3. In the three rows from top to bottom, 5, 10, and 15 innermost walls, respectively, are removed from the corresponding ZG MWCNTs (9,0)/(18,0)/.../(9*n*,0). In each row from left to right, the numbers of walls left in the MWCNTs are 5, 10, 15, 20, and 25.

Prior to structural optimization, the radii of the innermost walls are 2.1, 3.8, and 5.6 nm for 5-wall, 10-wall, and 15-wall removed MWCNTs, respectively. As shown in Figure 3, upon energy relaxation, the cross-sections of all the MWCNTs are transformed from circular to polygonal shapes with flattened sides. The cross-sectional shapes in Figure 3h and 3m agree well with reported experimental results [35,52]. A general trend is observed: along each column from top to bottom or along each row from left to right, the configurations of the innermost walls deviate progressively further from the circular shape. These morphological evolutions are generally due to the competition of the inter-wall vdW energy release and the out-of-plane bending penalty, as discussed earlier. Along each column from top to bottom, with increasing radius of the innermost walls, the innermost walls and the whole MWCNTs are increasingly more complaint, and thus easier to bend to facilitate inter-wall vdW energy release. While along each row from left to right, with increasing wall numbers, the increasingly more excessive inter-wall vdW energy imposes progressively higher compressive stress onto the innermost walls, thereby inducing larger deformation from their circular shape.

## Conclusions

We have performed large-scale quasi-continuum simulations on the stable cross-sectional configurations of MWCNTs. Our simulations show that both the inter-wall spacing and the radius of the innermost wall play important roles in regulating the stable cross-sectional configurations of the MWCNTs. The relaxed cross-sectional configurations agree well with prior experimental observations. We attribute the shape transformations to the inter-wall vdW interaction energy release, penalized by the increase of the wall bending energy. This deformation mechanism is opposite to that in bent, twisted, and compressed MWCNTs, where the deformation morphologies such as wave-like periodic rippling and Yoshimura pattern are driven by the in-plane strain energy release, but penalized by the increase of the inter-wall vdW interaction energy [16-18].

It should be pointed out that from our simulations, the cross-sectional corrugations occur only for MWCNTs for which inter-wall vdW interactions are present. This may explain that cross-sectional corrugations were rarely reported for MWCNTs in previous all-atom simulations since such thick MWCNTs are typically beyond the access of all-atom simulations. Given the intimate coupling between the deformation morphologies and the electrical/magnetic properties of CNTs, the predicted stable cross-sectional configurations provide fundamental guidance to tailor the electrical properties of MWCNTs.
